# Comparison of the Allergenicity and Immunogenicity of Camel and Cow’s Milk—A Study in Brown Norway Rats

**DOI:** 10.3390/nu10121903

**Published:** 2018-12-04

**Authors:** Natalia Zofia Maryniak, Egon Bech Hansen, Anne-Sofie Ravn Ballegaard, Ana Isabel Sancho, Katrine Lindholm Bøgh

**Affiliations:** Division of Diet, Disease Prevention and Toxicology, National Food Institute, Technical University of Denmark, 2800 Kgs. Lyngby, Denmark; nazoma@food.dtu.dk (N.Z.M.); egbh@food.dtu.dk (E.B.H.); anravn@food.dtu.dk (A.-S.R.B.), anasa@food.dtu.dk (A.I.S.)

**Keywords:** food allergy, cow’s milk, camel milk, infant formula, animal models

## Abstract

Background: When breastfeeding is impossible or insufficient, the use of cow’s milk-based hypoallergenic infant formulas is an option for infants suffering from or at risk of developing cow’s milk allergy. As the Camelidae family has a large evolutionary distance to the Bovidae family and as camel milk differs from cow’s milk protein composition, there is a growing interest in investigating the suitability of camel milk as an alternative to cow’s milk-based hypoallergenic infant formulas. Methods: The aim of the study was to compare the allergenicity and immunogenicity of camel and cow’s milk as well as investigating their cross-reactivity using a Brown Norway rat model. Rats were immunised intraperitoneally with one of four products: camel milk, cow’s milk, cow’s milk casein or cow’s milk whey fraction. Immunogenicity, sensitising capacity, antibody avidity and cross-reactivity were evaluated by means of different ELISAs. The eliciting capacity was evaluated by an ear swelling test. Results: Camel and cow’s milk showed similarity in their inherent immunogenicity, sensitising and eliciting capacity. Results show that there was a lower cross-reactivity between caseins than between whey proteins from camel and cow’s milk. Conclusions: The study showed that camel and cow’s milk have a low cross-reactivity, indicating a low protein similarity. Results demonstrate that camel milk could be a promising alternative to cow’s milk-based hypoallergenic infant formulas.

## 1. Introduction

Cow’s milk allergy (CMA) is the most prevalent food allergy in infants and small children [1], affecting around 2.5% [2,3], although differences are observed between studies and countries [4]. Although most CMA children outgrow their allergy, some keep it for life [5]. Originally, it was though that most children did outgrow their CMA before the age of three years, but there seems to be a tendency that more and more children outgrow their CMA later in life and for some it may even last for lifetime [6,7]. Breastfeeding is the most suited source of nutrition for a newborn infant [8]. However, in some situations, breastfeeding is impossible or insufficient and a substitute such as an infant formula is needed [9]. Infant formulas are usually based on cow’s milk, as this is the most easily accessible milk source globally [10]. When an infant suffers from or is at risk of developing CMA, alternatives to conventional infant formulas are recommended such as hypoallergenic infant formulas, based on extensively or partially hydrolysed cow’s milk proteins [11]. In addition to cow’s milk-based hypoallergenic infant formulas, additional alternatives to conventional infant formulas are found on the market, such as amino acid-based infant formulas, plant-based infant formulas (e.g., soya-based) and infant formulas based on other mammalian milk (e.g., goat or sheep) [8,12,13]. Extensively and partially hydrolysed infant formulas as well as amino acid-based infant formulas are poor in flavour, and, thus, some newborns may refuse them [5,14]. On the other hand, it has been reported that sheep and goat milk-based infant formulas may only be an alternative for some newborns due to a high cross-reactivity between cow’s milk proteins and proteins from goat and sheep milk [13,15]. In addition, plant-based infant formulas are seldom recommended due to their low nutritional value [16,17]. For those reasons, new or improved alternatives to conventional infant formulas are still of interest.

Due to the large evolutionary distance between Camelus dromedaries (Camelidae family) and the Bovidae family animals, camel milk is quite different in its composition compared to cow’s milk. Equivalent to human milk, the allergenic milk protein *β*-lactoglobulin (BLG) is also absent in camel milk [18]. Moreover, similar to human milk, camel milk has approximately double the amount of β-casein and approximately five times the amount of immunoglobulins in comparison to cow’s milk [19]. Rastani et al. [13] showed that CMA patients did not recognise camel milk by immunoblotting and concluded that camel milk is a promising alternative to cow’s milk for infant formula manufacture. Further, based on double-blind, placebo-controlled food challenges, Navarre-Rodriguez et al. [20] concluded that camel milk is a safe and tolerable alternative for CMA patients above the age of one year. Camel milk is already commercially available in the Middle East, Australia, United Kingdom and the Netherlands [21,22,23,24]. In other regions such as in African countries, it is a traditionally consumed product, although without a control on its quality and safety [25]. There are a number of studies showing that camel milk is nutritionally suitable for human consumption [21,26]. For those reasons, camel milk is an exciting and suitable product with the potential to be a future alternative to hypoallergenic cow’s milk-based infant formulas in prevention, treatment and management of CMA in infants and small children. 

The purpose of this study was to investigate the immunogenicity and allergenicity of camel and cow’s milk as well as studying cross-reactivity between proteins from the two sources. To do this, Brown Norway (BN) rats were immunised intraperitoneally (i.p.) with either camel milk, cow’s milk, cow’s milk casein fraction or cow’s milk whey fraction and antibody responses were evaluated for level, specificity, avidity, functionality and cross-reactivity by means of different enzyme-linked immunosorbent assays (ELISAs), immunoblotting and in vivo test. This should allow for an overview of the usability of camel milk as an alternative to hypoallergenic infant formulas. 

## 2. Materials and Methods

### 2.1. Products

Powders of cow’s milk, cow’s milk casein fraction and cow’s milk whey fraction were kindly provided by Arla Foods Ingredients Videbæk, Denmark. Powder of camel milk was kindly provided by Dairy Farm Smits, Berlicum, the Netherlands. Products were tested by Pierce™ LAL Chromogenic Endotoxin Quantitation Kit (88282, Thermo Fisher, Waltham, MA, USA) in accordance with the instruction given by the manufacturer. Whereas camel milk, cow’s milk and cow’s milk whey fraction had an endotoxin level <2 endotoxin units (EU) per mg of protein, cow’s milk casein fraction had an endotoxin level of approximately 66 EU per mg of protein. 

### 2.2. In Silico Protein Analyses

CLC Main Workbench 8.0 (Redwood City, CA, USA) was used to compare selected protein amino acid sequences from cow’s milk with those of goat, sheep, camel and human milk. Protein sequences were downloaded from UniProt (http://www.uniprot.org).

### 2.3. Denaturation of Products

Camel milk and cow’s milk were denatured to obtain unfolded structures of proteins. Denaturation was performed by reduction and alkylation, as previously described by Madsen et al. [27]. 

### 2.4. SDS-PAGE Electrophoresis

Sodium dodecyl sulphate-polyacrylamide gel electrophoresis (SDS-PAGE) with camel milk, denatured camel milk, cow’s milk, denatured cow’s milk, cow’s milk casein fraction and cow’s milk whey fraction was performed using 5 µg of each product dissolved in Laemmli buffer (65.8 mM Tris-HCl, pH 6.8, 26.3% (*w*/*v*) glycerol and 2.1% (*w*/*v*) SDS, 161-0737, Bio-Rad, Hercules, CA, USA) with addition of *β*-mercaptoethanol (14.2 M, 161-0710, Bio-Rad). Samples were incubated for 5 min at 95 °C and afterwards loaded onto a 4–20% gel (Mini-Protean TGX Stain-Free gel, 456-8093, Bio-Rad). SDS-PAGE was performed in running buffer (25 mM Tris and 192 mM Glycine and with addition of 0.1% (*w*/*v*) SDS, pH 8.3, 161-0732, Bio-Rad). Additionally, 10 µL of the molecular weight Precision Plus Protein™ Unstained Standard (161-0363, Bio-Rad) was loaded onto the gel. Gel electrophoresis was run at 200 V with constant current at room temperature (RT). Afterwards, the gel was stained with Bio Safe™ Coomassie (161-0786, Bio-Rad) for 1 h at RT and photographed using Imager ChemiDoc XRS+ (Bio-Rad). 

### 2.5. Animals

BN rats were from the in-house breeding colony, at the National Food Institute, Technical University of Denmark, Denmark, and kept in macrolon cages at 22 °C ± 1 °C with 55 ± 5% relative humidity at a 12-h light–dark cycle. Air exchange was applied 8–10 times per hour with overpressure. BN rats were inspected twice a day and weighted once per week. Rats were kept on a diet free from milk and soy allergens for ≥10 generations. Feed containing rice flour and fish was given ad libitum as well as was acidified tap water. 

### 2.6. Animal Sensitisation Studies

**Figure 1:**
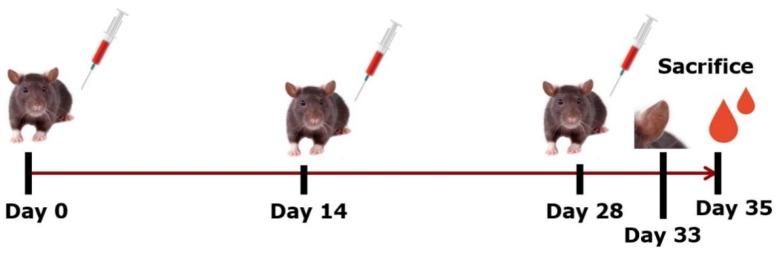
Animal experimental design. Brown Norway rats were immunised i.p. with 200 µg of camel milk, cow’s milk, cow’s milk casein fraction or cow’s milk whey fraction three times, at Days 0, 14 and 28. At Day 33 an ear swelling test was performed and at Day 35 rats were sacrificed and blood collected. Pictures were purchased from https://www.colourbox.com.

To sensitise animals and raise antibodies against camel milk, cow’s milk, cow’s milk casein fraction or cow’s milk whey fraction, BN rats 4–7 weeks of age, were divided into five groups of eight rats (*n* = four/gender), and housed two per cage. Groups of rats were immunised i.p. three times with 200 µg of product dissolved in phosphate buffer saline (PBS) (137 mM NaCl, 3 mM KCl, 8 mM Na_2_HPO_4_, 1 mM KH_2_PO_4_, pH 7.2) without the use of adjuvant one time at Day 0, 14 and 28 (Figure 1). One group of rats was not immunised to act as a control group (naïve animals) for an ear swelling test. At Day 35, rats were sacrificed and blood collected. The animal experiment was carried out at the National Food Institute, Technical University of Denmark under ethical approval given by the Danish Animal Experiments Inspectorate and the authorisation number 2015-15-0201-00553-C1. The experiment was overseen by the National Food Institute’s in-house Animal Welfare Committee for animal care and use. 

### 2.7. Ear Swelling Test

To investigate the eliciting capacity of camel and cow’s milk, at Day 33 of the experiment, an ear swelling test was performed. Rats were anesthetised with hypnorm-dormicum and baseline ear thickness was measured. Subsequently, 20 µL of PBS with 10 µg of camel milk or 10 µg of cow’s milk were injected into the right or left ear, respectively, and ear thicknesses were measured again one hour after injections. Naïve rats were included to see unspecific ear swelling and irritation capacity after camel and cow’s milk protein ear injection. Delta ear swelling was calculated. 

### 2.8. Indirect ELISA for Specific IgG1 Detection

To detect IgG1 antibodies specific for camel milk, denatured camel milk, cow’s milk and denatured cow’s milk, indirect ELISAs were performed using Maxisorp microtitre plates (96-well, Nunc, Roskilde, Denmark). Plates were coated with 100 µL/well of 10 µg/mL of camel milk, denatured camel milk, cow’s milk or denatured cow’s milk, in coating buffer (15 mM Na_2_CO_3_, 35 mM NaHCO_3_, pH 9.6), and incubated overnight at 4 °C. Between each step, plates were washed five times in PBS with 0.01% (*w*/*v*) Tween 20 (PBS-T). For all steps that required incubation, plates were incubated for one hour in the dark at RT, with gentle agitation. First, plates were incubated with 50 µL/well of two-fold serial dilution of serum samples (*v*/*v*) in PBS-T. In each plate, positive and negative control serum samples were included in order to identify potential plate-to-plate variance. For antibody detection, 50 µL/well of secondary antibody (horse radish peroxidase (HRP)-labelled-mouse-anti-rat IgG1, 3060-05, Southern Biotech, Birmingham, AL, USA) diluted 1:20,000 (*v*/*v*) in PBS-T was added to the plates. After incubation plates were additionally washed twice with tap water. To visualise specific antibody detection, 100 µL/well of TMB-one (3,3′,5,5′-tetramethylobenzidine, 4380A, Kementec Diagnosis, Taastrup, Denmark) was added and incubated for 12 min at RT. The reaction was stopped with 100 µL/well 0.2 M H_2_SO_4_ and the absorbance was measured at 450 nm with a reference wavelength of 630 nm using a microtitre reader (Gen5, BioTek, EL800 Instrument, Winooski, VT, USA). The cut-off values were set to be higher than the mean absorbance of negative control plus three times the standard deviation (SD). Results were expressed in log2 titre values with a cut-off at the optical density (OD) of 0.1 for IgG1 specific for camel milk, cow’s milk and denatured cow’s milk and 0.15 for IgG1 specific for denatured camel milk.

### 2.9. Antibody Capture ELISA to Detect Specific IgE

To detect IgE specific for camel milk, denatured camel milk, cow’s milk and denatured cow’s milk, antibody capture ELISAs were performed using Maxisorp microtitre plates (96-well, Nunc) coated with 100 µL/well of mouse anti-rat IgE (HDMAB-123, Hydri-Domus, Nottingham, UK) diluted 1:2000 in coating buffer and incubated overnight at 4 °C. Between each step, plates were washed five times with PBS-T. For all steps that required incubation, plates were incubated for one hour in the dark at RT, with gentle agitation. For camel and cow’s milk specific IgE detection, antibody capture ELISA was optimised to use proper blocking for each product. Plates were blocked at 37 °C, 200 µL/well, with 3% (*v*/*v*) horse serum for camel milk specific IgE detection and 5% (*v*/*v*) rabbit serum for cow’s milk specific IgE detection, diluted in PBS-T. Subsequently, plates were incubated for one hour with 50 µL/well of two-fold serial dilution of serum samples (*v*/*v*) in PBS-T. In each plate, positive and negative control serum samples were included. Afterwards, 50 µL/well of 0.05 µg/mL of 10:1 digoxigenin (DIG)-coupled camel milk or 0.1 µg/mL of 10:1 DIG-coupled cow’s milk in PBS-T were added, to detect specific IgE. Next, plates were incubated with 100 µL/well of HRP-labelled sheep-anti-DIG-POD (11633716001, Roche, Diagnostics GmbH, Mannheim, Germany) diluted 1:1000 (*v*/*v*) in PBS-T. After this step, plates were additionally washed twice with tap water and incubated for 12 min with 100 µL/well of TMB-one (Kementec Diagnosis). The reaction was stopped with 100 µL/well of 0.2 M H_2_SO_4_ and the absorbance was measured. Results were expressed as log2 titre value with an individual cut-off of plates at an OD of 0.145–0.2 for IgE specific for camel milk and of 0.125–0.175 for IgE specific for cow’s milk.

### 2.10. Avidity Measurements

To measure binding strength between antigens and IgG1 antibodies from serum samples, avidity ELISA was performed as previously described by Bøgh et al. [28]. Serum samples from rats that reached an OD of at least 0.5 were included.

### 2.11. Inhibitory ELISA

To examine the cross-reactivity between proteins from camel and cow’s milk, inhibitory ELISA was performed. The procedure was as described for the indirect IgG1 ELISA with few exceptions. Serum samples for each group of animals were pooled and diluted in PBS-T to reach an OD of approximately 2.0. Serum pools were then pre-incubated for one hour with ten-fold serial dilutions of camel and cow’s milk. After pre-incubation, samples were added to the plates in duplicates and incubated for one hour. The assay was performed twice. The results were expressed in percentage inhibition against the concentration of the inhibitor.

### 2.12. Immunoblotting

To do immunoblotting, SDS-PAGE was performed with 5 µg of camel and cow’s milk as described previously. In addition, SDS-PAGE with an eight-time higher load of proteins (40 µg) was performed to visualise cross-reactivity. After SDS-PAGE, proteins were transferred onto polyvinylidene difluoride membranes (Trans-Blot^®^ Turbo™ Mini PVDF Transfer Pack, 1704156, Bio-Rad) by semidry blotting (Trans-Blot^®^ Turbo™ Transfer System, 170-4150, Bio-Rad) at constant 200 V. Membranes were washed three times for 5 min in PBS-T (0.05% *v*/*v* Tween 20) and each blocked with 20 mL of 5% ovalbumin (OVA, egg whites from chicken, Sigma Aldrich, St. Louis, MO, USA) diluted in PBS-T (0.1% *v*/*v* Tween 20) and incubated for one hour in the dark at RT, on a shaking table. The 5% OVA solution was used during the whole experiment as a blocking solution. After blocking, membrane was divided into two pieces, both pieces with 5 µg of camel and cow’s milk. Next, 10 mL of serum pooled from rats immunised with cow’s milk diluted 1:3000 (*v*/*v*) in blocking solution or serum pooled from rats immunised with camel milk diluted 1:8000 (*v*/*v*) in blocking solution were added separately to each half of the membrane containing 5 µg of camel and cow’s milk and incubated for one hour in the dark at RT, on a shaking table. Half of the membrane with 40 µg of cow’s milk was incubated with serum pooled from rats immunised with camel milk diluted 1:500 (*v*/*v*) in blocking solution, while the other half of the membrane with 40 µg of camel milk was incubated with serum pooled from rats immunised with cow’s milk diluted 1:500 (*v*/*v*) in blocking solution. Afterwards, membranes were washed three times for 5 min in PBS-T (0.05% *v*/*v* Tween 20) and 10 mL of the secondary antibody diluted 1:15,000 together with StrepTacin-HRP conjugate (Bio-Rad) for Precision Plus Protein™ Unstained Standard detection, diluted 1:15,000 in blocking solution were added to each half of the membrane. Membranes were incubated for one hour in the dark at RT, on a shaking table. Subsequently, membranes were washed three times for 5 min in PBS-T (0.05% *v*/*v* Tween 20) followed by PBS washing two times for 5 min to remove the detergent. Membranes were incubated with peroxidase substrate (Clarity™ Western ECL Substrate, 1705060, Bio-Rad) for 5 min. After incubation, membranes were developed and photographed using Imager ChemiDoc XRS+ (Bio-Rad).

### 2.13. Statistical Analysis of Data 

Graphs and statistical analyses of the data were performed using GraphPrism version 7.0 (San Diego, CA, USA). Results from indirect and antibody-capture ELISAs were expressed as log2 antibody titre values.

ELISA results expressed as log2 antibody titres were tested for normality distribution. Based on the results, either parametric or non-parametric *t*-tests were performed. Differences were regarded as statistically significant when *p* ≤ 0.05. Asterisks indicate statistically significant differences between two given groups: ∗ = *p* ≤ 0.05, ∗∗ = *p* ≤ 0.01, ∗∗∗ = *p* ≤ 0.001, ∗∗∗∗ *p* ≤ 0.0001. 

Inhibition curves resulting from avidity and inhibitory ELISA were examined with one-way repeated-measurements ANOVA test. Analyses showed no statistically significant differences between curves, thus IC_50_ calculations were performed. IC_50_ was calculated using sigmoidal dose response with non-linear regression.

## 3. Results

### 3.1. Protein Characterisation

The primary sequence from selected cow’s milk proteins were aligned to their counterpart proteins in milk from goat, sheep, camel and human to investigate the amino acid sequence identity between the different species and to predict the potential cross-reactivity between proteins of interest. As shown in Table 1, goat and sheep milk protein sequences show a very high percentage identity to cow’s milk proteins, ranging from a protein sequence identity of 85–95% for goat and sheep. A much lower protein sequence identity was evidenced between camel and cow’s milk proteins, where the protein identity ranged from 47% to 81%. This is very similar to the protein sequence identity of human and cow’s milk proteins ranging from 33% to 76% and human and camel milk proteins ranking from 40% to 76%. In addition, neither camel nor human milk contains BLG [18]. Similarities between camel and cow’s milk caseins sequences were shown to be slightly lower than between the whey proteins. 

**Figure 2:**
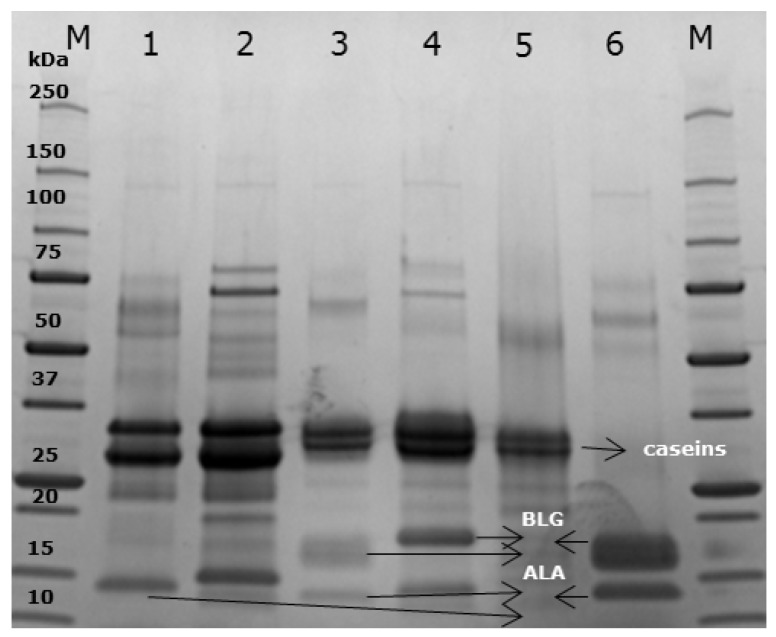
SDS-PAGE electrophoresis. Gel electrophoresis, with native and denatured camel and cow’s milk as well as with native cow’s milk casein fraction and native cow’s milk whey fraction, was performed to display protein profiles. M, protein standard (kDa); 1, camel milk; 2, denatured camel milk; 3, cow’s milk; 4, denatured cow’s milk; 5, cow’s milk casein fraction; 6, cow’s milk whey fraction. BLG, *β*-lactoglobulin; ALA, *α*-lactalbumin.

SDS-PAGE electrophoresis was performed to display the protein profile of the products used in this study. Caseins run as thick bands between 25 and 37 kilodalton (kDa) in both camel and cow’s milk (Lanes 1–4, Figure 2) as well as in the casein fraction of cow’s milk (Lane 5) [31]. The band corresponding to a molecular weight (MW) of around 30 kDa represents *β*-casein while the band immediately above represents *α*-caseins with a MW of around 35 kDa [19,31]. In cow’s milk as well as in the whey fraction of cow’s milk (Lanes 3 and 6), a clear band representing BLG is evident (~18 kDa) [1], which is not present in camel milk. In all lanes except for the lane corresponding to the casein fraction of cow’s milk (Lane 6), the lower band represents *α*-lactalbumin (ALA) (~14 kDa), while the two upper bands most likely represent lactoferrin (LF) (~75 kDa) and serum albumin (SA) (~66 kDa) [19]. Immunoglobulins (~150 kDa) are only hardly seen due to their low amount in the milk products. LF and SA are slightly more visible in the denatured version of the milk products (Lanes 2 and 4) than their native counterparts. Another difference between the native and denatured version of the milk products are a lower mobility of proteins in the denatured versions compared to the native versions. 

### 3.2. Camel and Cow’s Milk Immunogenicity and Cross-Reactivity

**Figure 3:**
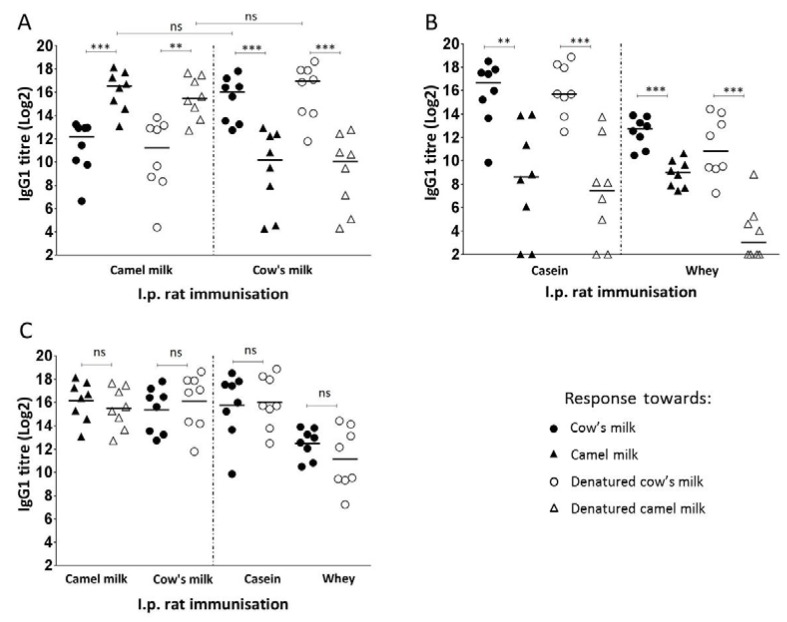
Specific IgG1 antibody responses. Comparison of specific IgG1 antibody responses toward cow’s milk (●), camel milk (▲), denatured cow’s milk (○) and denatured camel milk (∆) raised in rats immunised with camel milk, cow’s milk, cow’s milk casein fraction or cow’s milk whey fraction. Each symbol represents the specific IgG1 titre value for an individual rat. (**A**) Comparison of native and denatured camel milk and cow’s milk specific IgG1 antibody responses in rats immunised with camel or cow’s milk, respectively. Horizontal lines display the median values for each group of rats. Statistically significant difference between two groups was determined using the non-parametric Mann–Whitney test. Asterisks indicate statistically significant differences between the two given groups when: ∗ = *p* ≤ 0.05, ∗∗ = *p* ≤ 0.01, ∗∗∗ = *p* ≤ 0.001, ∗∗∗∗ *p* ≤ 0.0001. (**B**) Comparison of native and denatured camel milk and cow’s milk specific IgG1 antibody responses in rats immunised with cow’s milk casein or whey fraction. Horizontal lines display the median values for each group of rats. Statistically significant difference between two groups was determined using the non-parametric Mann–Whitney test. Asterisks indicate statistically significant differences between the two given groups when: ∗ = *p* ≤ 0.05, ∗∗ = *p* ≤ 0.01, ∗∗∗ = *p* ≤ 0.001, ∗∗∗∗ *p* ≤ 0.0001. (**C**) Comparison of IgG1 antibody reactivity against native vs. denatured camel and cow’s milk. Horizontal lines display the mean values for each group of rats. Statistically significant difference between two groups was determined using the parametric *t*-test. Asterisks indicate statistically significant differences between the two given groups when: ∗ = *p* ≤ 0.05, ∗∗ = *p* ≤ 0.01, ∗∗∗ = *p* ≤ 0.001, ∗∗∗∗ *p* ≤ 0.0001.

Serum samples from individual BN rats immunised with camel milk, cow’s milk, cow’s milk casein fraction or cow’s milk whey fraction were assessed for specific IgG1 by means of indirect ELISAs. Figure 3A shows the IgG1 responses against both the native and denatured version of camel as well as cow’s milk proteins.

The immunogenicity of camel and cow’s milk appears to be very similar as there is no statistically significant difference between the level of camel milk specific IgG1 raised against camel milk and the level of cow’s milk specific IgG1 raised against cow’s milk (Figure 3A). For both antibodies raised against camel or cow’s milk proteins, there is a statistically significant difference between the IgG1 reactivity towards camel milk and cow’s milk proteins, indicating a low cross-reactivity between camel and cow’s milk proteins. For antibodies raised against camel milk, the IgG1 reactivity against cow’s milk proteins was ~30 fold lower than the reactivity against camel milk proteins, measured by the amount of specific antibodies. Opposite the IgG1 reactivity against camel milk proteins was ~50-fold lower than the reactivity against cow’s milk proteins for sera raised against cow’s milk proteins. This was shown irrespectively of responses that were measured against the native or denatured version of the milk proteins.

The IgG1 responses in rats immunised with either the casein or the whey fraction of cow’s milk, are shown in Figure 3B. For both groups of animals, the IgG1 responses against native and denatured camel milk were statistically significantly lower than the responses against native and denatured cow’s milk, stressing a low cross-reactivity for both the casein and the whey fraction of camel and cow’s milk proteins. For antibodies raised against casein, the IgG1 reactivity against native camel milk proteins was ~250-fold lower than the reactivity against cow’s milk proteins, while for antibodies raised against whey, the IgG1 reactivity against camel milk proteins was ~15-fold lower than the reactivity against cow’s milk proteins. This indicates a lower cross-reactivity between camel and cow’s milk caseins than whey proteins.

### 3.3. Linear and Conformational Epitope Recognition

The study showed that there were no statistically significant differences between the IgG1 responses against the native and denatured versions of milk proteins for rats immunised with neither camel milk nor cow’s milk, indicating that linear epitopes are dominating both responses (Figure 3C). In addition, the IgG1 raised against cow’s milk caseins showed no statistically significant difference in their reactivity against the native or denatured version of milk proteins with an approximate ratio of 1:1. In contrast, although IgG1 raised against cow’s milk whey showed no statistically significant difference in their reactivity against the native and denatured version of milk proteins, the ratio between IgG1 specific for native vs. denatured cow’s milk was 4:1. This demonstrates that, while caseins primarily induce antibodies against linear epitopes, whey primarily induces antibodies against conformational epitopes. 

### 3.4. Inhibitory ELISA

Inhibitory ELISA was performed with sera pools from groups of rats immunised with camel milk, cow’s milk, cow’s milk casein or whey fraction in order to evaluate the competitive capacity of native as well as denatured camel and cow’s milk. 

#### 3.4.1. IgG1 Antibody Competition of Native Camel and Cow’s Milk

**Figure 4:**
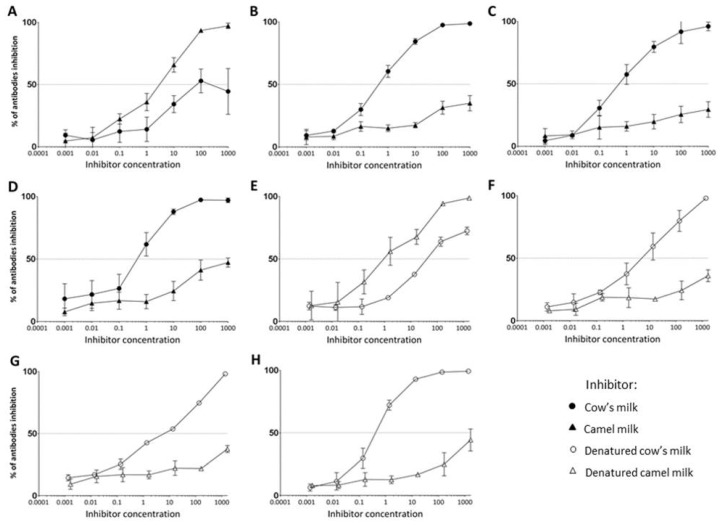
IgG1 antibody binding competition. Inhibitory ELISA with native (●) or denatured (**○**) cow’s milk or native (▲) or denatured (∆) camel milk as inhibitors was performed using serum pools from rats immunised with camel milk, cow’s milk, cow’s milk casein fraction or cow’s milk whey fraction. Each symbol represents the percent inhibition of IgG1 specific antibodies at different inhibitor concentrations. Error bars in the inhibition curves represent ± standard deviation (SD). (**A**) Inhibition curve for sera raised against camel milk. (**B**) Inhibition curve for sera raised against cow’s milk. (**C**) Inhibition curve for sera raised against cow’s milk casein fraction. (**D**) Inhibition curve for sera raised against cow’s milk whey fraction. (**E**) Inhibition curve for sera raised against linear epitopes of camel milk. (**F**) Inhibition curve for sera raised against linear epitopes of cow’s milk. (**G**) Inhibition curve for sera raised against linear epitopes of cow’s milk casein fraction. (**H**) Inhibition curve for sera raised against linear epitopes of cow’s milk whey fraction.

While native camel milk was able to fully inhibit antibodies raised against camel milk, native cow’s milk was only able to inhibit ~50% of the antibodies raised against camel milk (Figure 4A). On the other hand, while native cow’s milk was fully capable of inhibiting the antibodies raised against cow’s milk, native camel milk was only capable of inhibiting ~35% of antibodies raised against cow’s milk (Figure 4B). While native cow’s milk was able to fully inhibit antibodies raised against both the casein and the whey fraction of cow’s milk, native camel milk was only able to inhibit ~30% of antibodies raised against cow’s milk casein (Figure 4C) and ~45% of antibodies raised against cow’s milk whey (Figure 4D). This confirms previous results, showing a lower cross-reactivity between casein compared to the whey fraction of camel and cow’s milk.

#### 3.4.2. IgG1 Antibody Competition Towards Denatured Camel and Cow’s Milk

By performing inhibitory ELISA with the use of denatured versions of camel and cow’s milk, we could only study the cross-reactivity as a measure of antibodies raised against linear epitopes. While denatured camel milk was able to inhibit fully antibodies raised against linear epitopes of camel milk, denatured cow’s milk was able to inhibit ~70% of the antibodies raised against linear epitopes of camel milk (Figure 4E). On the other hand, while denatured cow’s milk was fully capable of inhibiting the antibodies raised against linear epitopes of cow’s milk, denatured camel milk was only capable of inhibiting ~35% of antibodies raised against cow’s milk (Figure 4F). While denatured cow’s milk was able to fully inhibit antibodies raised against both linear epitopes of the casein and the whey fraction of cow’s milk, denatured camel milk was only able to inhibit ~35% of antibodies raised against linear epitopes of cow’s milk casein (Figure 4G) and ~45% of antibodies raised against linear epitopes of cow’s milk whey (Figure 4H). This indicated a slightly higher cross-reactivity between linear epitopes compared to conformational epitopes of camel and cow’s milk.

### 3.5. Specific IgG1 Antibody Avidity

**Figure 5:**
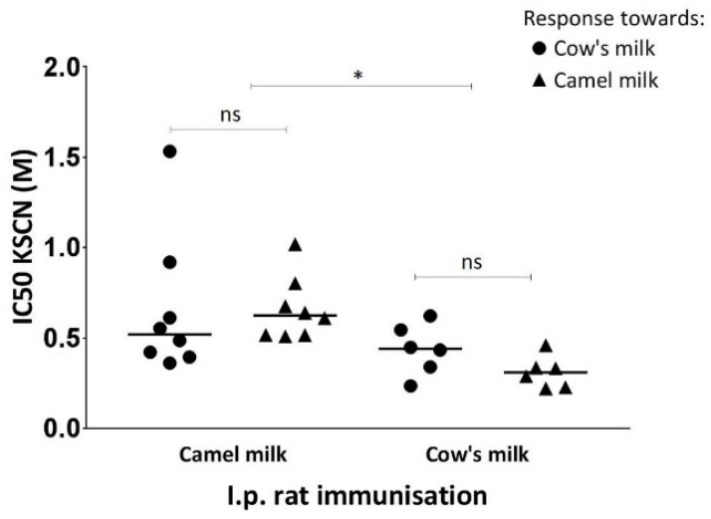
Avidity of IgG1 specific for cow’s milk (●) or camel milk (▲). Serum samples from rats immunised with camel milk or cow’s milk were evaluated to compare specific IgG1 antibody binding strength towards camel or cow’s milk. Each symbol represents an individual rat. The avidity is expressed as potassium thiocyanate concentration needed to inhibit 50% of the IgG1 response towards camel or cow’s milk for groups of rats immunised with camel milk or cow’s milk. Horizontal lines display the median values for each group of rats. Statistically significant difference between two groups was determined using the non-parametric Mann–Whitney test. Asterisks indicate statistically significant differences between two given groups when: ∗ = *p* ≤ 0.05, ∗∗ = *p* ≤ 0.01, ∗∗∗ = *p* ≤ 0.001, ∗∗∗∗ *p* ≤ 0.0001.

Avidity ELISAs were performed to evaluate binding strength between specific IgG1 antibodies and the milk proteins. Figure 5 displays the amount of potassium thiocyanate (KSCN) needed to inhibit 50% of the antibody–antigen binding. Results indicated that there were no statistically significant differences in binding strength between IgG1 raised against camel milk and camel milk or cow’s milk. Similar results were shown for IgG1 raised against cow’s milk and their binding strength towards cow’s milk and camel milk, although slightly higher avidity was shown between antibodies raised against cow’s milk and cow’s milk compared to the avidity between antibodies raised against cow’s milk and camel milk.

### 3.6. Sensitising Capacity of Camel and Cow’s Milk

**Figure 6:**
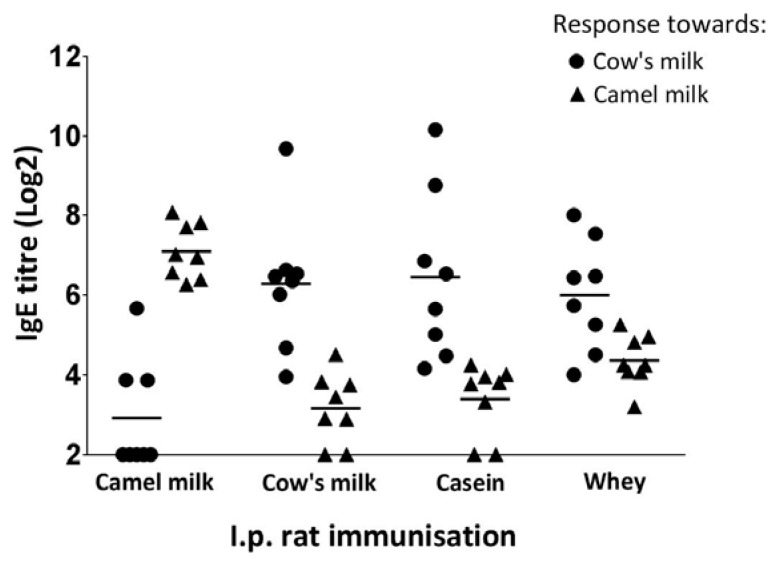
Specific IgE antibody responses. Comparison of specific IgE responses towards cow’s (●) and camel milk (▲) in rats immunised with camel milk, cow’s milk, cow’s milk casein fraction or cow’s milk whey fraction. Each symbol represents a specific IgE titre value for an individual rat. Horizontal lines on the graph display the median values for each group of rats.

As IgE is the main player in food allergies [32], specific IgE titres were determined by the use of antibody-capture ELISAs. The results showed no obvious differences in the sensitising capacity of camel and cow’s milk proteins, both products containing the capacity to induce high levels of specific IgE antibodies (Figure 6). No statistical analysis could be performed as the camel and cow’s milk assays cannot be directly compared because of their potential different sensitivity. In line with the specific IgG1 responses, also for the specific IgE responses a low cross-reactivity between camel and cow’s milk proteins was identified. Furthermore, in accordance with the IgG1 results, also for the IgE results a lower cross-reactivity could be observed for the casein fraction compared to the whey fraction of camel and cow’s milk proteins.

### 3.7. Eliciting Capacity of Camel and Cow’s Milk

**Figure 7:**
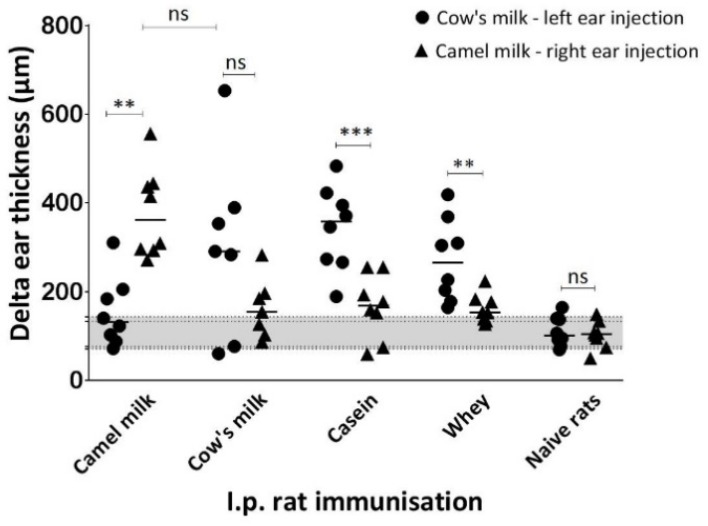
IgE functionality. Comparison of eliciting capacity of camel and cow’s milk measured by an ear swelling test in rats immunised with camel milk, cow’s milk, cow’s milk casein fraction or cow’s milk whey fraction. Delta ear thicknesses was calculated based on differences in ear thickness before and one hour after the ear injection of cow’s milk solution to the left ear (●) and camel milk solution to the right ear (▲) at Day 33. Each symbol represents the delta ear thickness for an individual rat. Horizontal lines on the graph display the median values for each group of rats. Naïve rats correspond to the control group and define the median delta ear thickness with SD (grey coloured area) indicating no elicitation but an ear swelling caused by the injection volume. Statistically significant difference between two groups was determined using the non-parametric Mann–Whitney test. Asterisks indicate statistically significant differences between two given groups when: ∗ = *p* ≤ 0.05, ∗∗ = *p* ≤ 0.01, ∗∗∗ = *p* ≤ 0.001, ∗∗∗∗ *p* ≤ 0.0001.

The ability of camel and cow’s milk to elicit allergic reactions was determined by an ear swelling test (Figure 7). Rats sensitised to camel milk showed a larger reaction towards camel milk than towards cow’s milk, and opposite rats sensitised to cow’s milk showed a larger reaction against cow’s milk than camel milk, which correlates very well with the specific IgE responses (Figure 6), and confirms the low cross-reactivity between camel and cow’s milk proteins. While a statistically significant difference was obtained for rats sensitised with camel milk, no statistically significant difference was obtained for rats sensitised with cow’s milk. This may be explained by the fact that only seven animals are included in the cow’s milk sensitised group compared to eight animals in the camel milk sensitised group, as one cow’s milk sensitised animal died during the ear swelling test due to anaphylaxis. This has biased the results as this animal would probably be the one that would have responded with the greatest ear swelling. The groups immunised with cow’s milk casein fraction or cow’s milk whey fraction both showed a significantly larger response towards cow’s milk compared to camel milk; however, in line with the antibody responses, the casein proteins were shown to have a lower cross-reactivity than the whey proteins. 

### 3.8. Immunoblot

**Figure 8:**
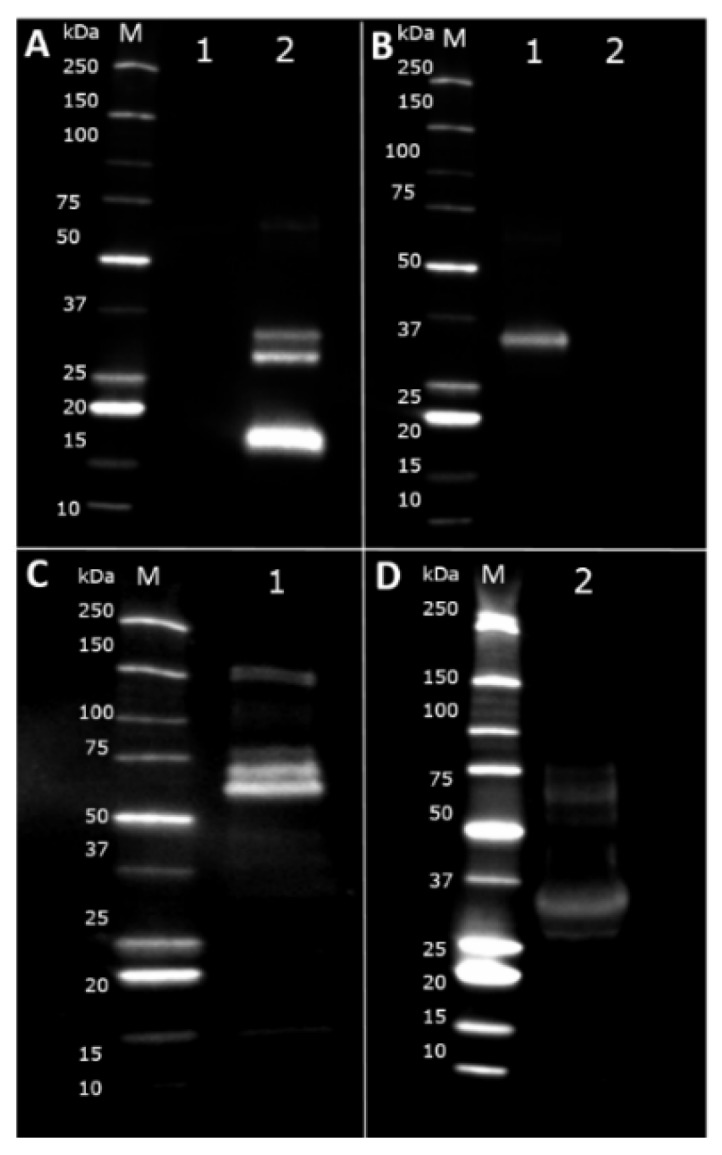
Immonublotting with camel and cow’s milk. M, 2 µL of Protein Standard (kDa); 1, camel milk; 2, cow’s milk. (**A**) Comparison of the reactivity of IgG1 antibodies specific for cow’s milk diluted 1:3000 (*v*/*v*) towards 5 µg of camel milk and cow’s milk (**B**) Comparison of the reactivity of IgG1 antibodies specific for camel milk diluted 1:8000 (*v*/*v*) towards 5 µg of camel milk and cow’s milk. (**C**) Cross-reactivity between IgG1 antibodies raised towards cow’s milk diluted 1:500 (*v*/*v*) and 40 µg of camel milk proteins. (**D**) Cross-reactivity between IgG1 antibodies raised towards camel milk diluted 1:500 (*v*/*v*) and 40 µg of cow’s milk proteins.

Immunoblotting was performed to investigate the specificity of the responses towards camel and cow’s milk. Figure 8A shows the specificity of antibodies raised against cow’s milk. BLG (~18 kDa) and the two casein fractions between 25 and 37 kDa were the proteins that antibodies specific for cow’s milk reacted most pronounced to. Moreover, a hardly visible band was seen between 50 and 75 kDa indicating a weak reactivity towards SA (~66 kDa). There was no visible reaction of antibodies specific for cow’s milk for camel milk proteins. The opposite situation is shown in Figure 8B where the specificity of antibodies raised against camel milk was evaluated. Here, antibodies reacted most pronounced with the camel milk *β*-casein fraction seen between 25 and 37 kDa standard marker bands, while there was no detectable reaction towards cow’s milk proteins. However, the pooled serum dilutions used for the immunoblots were high, with a dilution of 1:3000 for sera raised against cow’s milk and 1:8000 for sera raised against camel milk, for which reasons only the proteins with the strongest IgG1 binding capacity were visualised. As the ELISA assay showed very low cross-reactivity between camel and cow’s milk proteins, we decided to use eight times higher protein concentration and lower serum pools dilution in order to visualise the proteins responsible for the cross-reactivity. The dilution used for both camel and cow’s milk raised sera was 1:500. Figure 8C,D show cross-reactivity between camel and cow’s milk proteins. Antibodies specific for cow’s milk were able to cross-react exclusively with camel milk whey proteins. There were visible bands between 50 and 75 kDa indicating the most pronounced cross-reactivity with camel milk SA (~66 kDa) and LF (~75 kDa). Another weak but visible band was detected around 15 kDa indicating a very low cross-reactivity with camel milk ALA (Figure 8C). Another very weakly detectable band was at approximately 150 kDa. This probably corresponded to immunoglobulins [33]. Antibodies specific for camel milk showed a very weak reaction with cow’s milk caseins between the 25 and 37 kDa standard marker bands, and with a whey protein appeared between the 50 and 75 kDa standard marker bands (Figure 8D).

## 4. Discussion

CMA is a major health issue of growing concern, for which reason the World Health Organisation (WHO) has created a guideline for diagnosis and rationale action [4]. Special hypoallergenic infant formulas for CMA infants as well as for infants in risk of developing CMA are available. These infant formulas are based on hydrolysed cow’s milk proteins and designated extensively and partially hydrolysed infant formulas, respectively, depending on the degree of hydrolysis and peptide size distribution profile. Additional formulas, based on plants or milk from other mammalians have also been suggested for CMA infants [8]. However, for example, goat and sheep milk cannot be recommended for all CMA infants due to the high protein homology and consequently high cross-reactivity with cow’s milk proteins [2,4,12]. One-humped camel—*Camelus dromedaries* (Camelidae family)—has a great evolutionary distance to animals from the Bovidae family [34]. Evolutionary distance directly influences milk protein composition variances, suggesting great differences between camel and cow’s milk. Having a different protein composition, camel milk is anticipated to be a suitable alternative to hypoallergenic cow’s milk-based infant formulas in the near future. To confirm a role for camel milk in management, primary prevention, and treatment of CMA, a combination of animal and human studies is needed. Using a BN rat model, we have compared immunogenicity, allergenicity, and cross-reactivity of camel and cow’s milk proteins.

The present study showed that camel and cow’s milk contain similar immunogenicity as well as allergenicity, being able to induce comparable levels of specific IgG1 and IgE antibodies with similar avidity. In addition, the eliciting capacity of the two milk products was shown to be similar. However, evaluation of the specific antibody reactivity towards cross-reactive proteins was low. 

Whereas antibody responses raised against caseins were dominated by epitopes of the linear type, antibody responses raised against whey proteins were dominated by conformational epitopes. This is in line with a previous study showing that while caseins primarily raised antibodies towards linear epitopes, BLG and ALA primarily induced antibodies towards conformational epitopes, irrespectively of animals were dosed i.p. or orally [21]. I.p. dosing enables the immune system to recognise proteins in their native, undigested state. These results correlate very well to the structural folding of the proteins within the casein and whey fraction of milk, where caseins possess a flexible unstructured folding [21,35,36], while the predominant proteins within whey, BLG and ALA are globular proteins containing two and four disulphide bonds, respectively [19,21,37].

Camel and cow’s milk proteins were in general shown to have a very low cross-reactivity. While approximately only 1 in 30 IgG1 antibodies raised against camel milk could react with cow’s milk, only approximately 1 in 50 IgG1 antibodies raised against cow’s milk could react with camel milk. The low cross-reactivity was confirmed by inhibitory ELISA where camel milk could only inhibit approximately 35% of the response against cow’s milk and cow’s milk could only inhibit approximately 50% of the response against camel milk. Similar results were observed for the IgE responses. Low cross-reactivity may reflect differences in the epitope pattern between camel and cow’s milk proteins directly correlated with a fairly low protein sequence identities.

The present study demonstrates that camel milk may be a suitable alternative to hypoallergenic infant formulas for CMA infant, as the low cross-reactivity should confer the camel milk low risk of inducing reactions. This is consistent with human studies showing that the introduction of camel milk to children with confirmed CMA, who did not respond to the conventional management, had a positive, rapid and long-lasting effect on their health [20,38]. Other studies have shown that neither camel milk caseins nor whey proteins could inhibit or bind to sera antibodies from patients with confirmed CMA [8,39]. In contrast to camel milk, both goat and sheep milk show a large cross-reactivity to cow’s milk [2,14,40], which is also reflected by the high protein identity, causing a similar epitope pattern. Human studies also showed that children with confirmed CMA reacted with goat milk due to IgE antibody cross-reactions [2,15]. In general, goat milk is not recommended for CMA patients without restrictive supervision of specialists [2,14].

The study showed that cow’s milk was more efficient in inhibiting binding to antibodies raised against camel milk than camel milk was in inhibiting binding to antibodies raised against cow’s milk. Certainly, the lack of BLG, one of the major allergenic proteins in cow’s milk [28], may at least partly explain this difference. This indicates that camel milk in general is a more suitable infant formula for CMA infant, than is cow’s milk for potential camel milk allergic infants.

The cross-reactivity between camel and cow’s milk caseins was found to be less than the cross-reactivity between camel and cow’s milk whey proteins, indicating that camel milk would be a more suitable alternative to hypoallergenic infant formulas for casein allergic infants than for whey allergic infants. This corresponds very well to the protein identity within the casein fraction compared to the whey fraction. In addition, immunoblot confirmed that antibodies specific for cow’s milk were able to exclusively react with camel milk whey proteins, confirming a predominance of whey proteins cross-reactivity. The reactivity was mostly towards camel milk SA, which is a protein that is rarely detected to independently cause cow’s milk allergy, and mostly sensitise together with other milk allergens [41,42].

Small differences were seen between the cross-reactivity accounted for by linear epitopes in comparison to cross-reactivity accounted for by conformational epitopes, where this study indicated that there is a tendency to a lower cross-reactivity between conformational epitopes compared to linear epitopes.

The difference in titre values in each group of immunised rats could reflect weaker antibody binding due to imperfect matching epitopes or be due to a low a low amount of shared epitopes. It can be stressed that the second option is the most likely, as the avidity of the cross-reacting antibodies was equal to the avidity of total population of antibodies. 

Overall, it is suggested that approximately 35–40% sequence identity between allergens is adequate to induce IgE cross-reactive binding [43]. However, cross-reactions are unusual below 50% identity and mostly requires more than 70% identity [44]. We can therefore conclude that the low level of cross-reactivity found in the present study is at the expected level for proteins of an evolutionary distance around 60%. A lower cross-reactivity would probably require an even lower sequence homology, which again would require milk from an animal with even larger evolutionary distance to cows. An alternative approach would be to look for milk from animals with a shorter evolutionary distance to humans. 

## 5. Conclusions

This study showed that, although camel and cow’s milk display similar immunogenicity and allergenicity, cross-reactivity between their proteins is low. Moreover, selected protein sequence alignments showed lower protein sequence identity between camel and cow’s milk proteins in comparison to other mammalian milk proteins such as goat or sheep. With this study, we showed that camel milk is a promising alternative to hypoallergenic cow’s milk-based infant formulas. For further evaluation of camel milk and its usefulness as a suitable alternative for hypoallergenic cow’s milk-based infant formulas in prevention, treatment and management of CMA, studies including oral animal sensitisation, primary prevention and treatment should be performed. In addition, mechanistic studies, including in vivo analyses of IgE functionality after oral challenge as well as evaluation of cellular changes in the gastrointestinal tract, would be of a great importance.

## Figures and Tables

**Table 1:** Amino acid sequence identity between cow’s and goat, sheep, camel and human milk proteins.

		Goat	Sheep	Camel	Human ^(c)^
Casein	*β*-casein	91	91	67	55 (60)
	*α*s1-casein	88	88	47	33 (40)
	*α*s2-casein	88	89	56	NA ^(a)^
	*κ*-casein	85	85	58	52 (60)
Whey	*α*-lactalbumin	95	95	60	74 (62)
	*β-*lactoglobulin	93	93	NA ^(b)^	NA ^(b)^
	serum albumin	88	92	81	76 (76)
	lactoferrin	92	92	75	70 (74)

Sequence identity (%) between selected cow’s milk proteins and their counterpart milk proteins from goat, sheep, camel and human expressed in percentage. Sequence alignments were performed using CLC Main Workbench 8.0 and UniProt and NCBI database. NA: not available. (a) *α*s2-casein not identified in human milk [29,30]. (b) *β*-lactoglobulin not available in camel and cow’s milk [18]. (c) Numbers in brackets represents sequence identity between human and camel milk. Accession number: *β*-casein: Cow: AAA30431; Goat: AAA30906; Sheep: CAA56139; Camel: CDO50354; Human: AAC82978. *α*s1-casein: Cow: AAA30429; Goat: CAA51022; Sheep: AEN84772; Camel: O97943; Human: CAA55185. *α*s2-casein: Cow: NP_776953; Goat: CAC21704; Sheep: CAA26983; Camel: O97944. *κ*-casein: Cow: CAA33034; Goat: CAA43174; Sheep: NP_001009378; Camel: CCI79378; Human: CAA47048. *α*-lactalbumin: Cow: CAA29664; Goat: CAA28797; Sheep: CAA29665; Camel: P00710; Human: AAA60345. *β*-lactoglobulin: Cow: CAA32835; Goat: CAA79623; Sheep: CAA31305. serum albumin: Cow: CAA41735; Goat: XP_005681801; Sheep: CAA34903; Camel: XP_010981066; Human: AAN17825; lactoferrin: Cow: AAA30610; Goat: AAA97958; Sheep: ACT76166; Camel: CAB53387; Human AAA59511.

## Abbreviations

ALA	*α*-lactalbumin
BLG	*β*-lactoglobulin
BN	Brown Norway
CMA	Cow’s milk allergy
DIG	dioxigenin
ELISA	Enzyme-linked immunosorbent assay
EU	endotoxin units
HRP	horse-radish peroxidase
IC_50_	half minimum inhibitory concentration
i.p.	intraperitoneally
kDa	kilodalton
KSCN	potassium thiocyanate
LF	actoferrin
LP	lactoperoxidase
MW	molecular weight
NA	not available
OD	optical density
OVA	ovalbumin
PBS	phosphate buffered saline
PBT-T	phosphate buffered saline-tween
PAGE	polyacrylamide gel electrophoresis
PVDF	polyvinylidene difluoride
RT	room temperature
SA	serum albumin
SD	standard deviation
SDS	sodium dodecyl sulphate
TMB	3,3′,5,5′-tetramethylbenzidine
Tris	2-amino-2-(hydroxymethyl)-1,3-propenediol
WHO	World Health Organisation
